# BDE-47 and male infertility: integrated network toxicology, single-cell transcriptomics, and *in vitro* evidence of GSTM1 downregulation and ferroptosis-related molecular remodeling in Leydig cells

**DOI:** 10.3389/fimmu.2026.1956997

**Published:** 2026-09-21

**Authors:** Xinyao Zhu, Shuang Wang, Tao Li, Yuqi Li, Tao Zhou

**Affiliations:** 1 Department of Urology, Affiliated Hospital of Southwest Medical University, Luzhou, Sichuan, China; 2 Nursing Department, Santai Hospital Affiliated to North Sichuan Medical College, Mianyang, Sichuan, China; 3 Department of Urology, Santai Hospital Affiliated to North Sichuan Medical College, Mianyang, Sichuan, China; 4 Department of Andrology, Nanjing Drum Tower Hospital, The Affiliated Hospital of Nanjing University Medical School, Nanjing, Jiangsu, China

**Keywords:** BDE-47, ferroptosis-related molecular remodeling, GSTM1, male infertility, non-obstructive azoospermia

## Abstract

**Background:**

BDE-47 is a widely detected polybrominated diphenyl ether with potential male reproductive toxicity, but the key testicular cell populations and molecular basis remain unclear.

**Methods:**

We integrated network toxicology, single-cell transcriptomic data from BDE-47-exposed mouse testes and human idiopathic non-obstructive azoospermia (NOA), and bulk transcriptomic data. High-dimensional weighted gene co-expression network analysis, exploratory metacell-based feature prioritization with SHapley Additive exPlanations analysis, ferroptosis-driver gene signature scoring, exploratory immune deconvolution, molecular docking, 100-ns molecular dynamics simulation, and TM3 cell experiments were performed.

**Results:**

We identified 113 shared candidate genes derived from BDE-47-related and NOA-associated gene sets, mainly enriched in xenobiotic metabolism, glutathione metabolism, oxidative stress, ferroptosis, and reproductive endocrine pathways. Mouse and human single-cell analyses localized the computational candidate signature to Leydig cells as one of the enriched somatic cell populations. GSTM1 was selected as an exploratory candidate based on multialgorithm screening, relative SHAP contribution, expression consistency across evidence layers, and redox-detoxification biological plausibility; it was expressed at lower levels in human NOA Leydig cells and bulk testicular tissue. At the cell level, lower GSTM1 expression was associated with a higher ferroptosis-driver gene signature score. Molecular simulations suggested potential structural compatibility between BDE-47 and GSTM1. In TM3 cells, BDE-47 reduced cellular metabolic activity and GSTM1 protein expression, decreased Gpx4 and Slc7a11 mRNA expression, and increased Acsl4 mRNA expression; ferrostatin-1 partially attenuated these transcriptional changes.

**Conclusions:**

BDE-47-related molecular evidence and human NOA-associated evidence showed molecular convergence around GSTM1 and ferroptosis-related molecular features in a Leydig-cell context. In TM3 cells, BDE-47 exposure was accompanied by GSTM1 downregulation and ferroptosis-related transcriptional remodeling. These observations are hypothesis-generating and do not establish direct binding, GSTM1-mediated effects, ferroptotic cell death, or a causal relationship between BDE-47 exposure and human NOA.

## 1 Introduction

BDE-47, or 2,2′,4,4′-tetrabromodiphenyl ether, is one of the most frequently detected and extensively studied congeners of polybrominated diphenyl ether (PBDE) flame retardants. PBDEs were previously used in electronic products, construction materials, furniture, textiles, and other industrial and consumer products to reduce flammability. Because these compounds are not chemically bound to product matrices, they can be gradually released into the surrounding environment during manufacturing, use, aging, and disposal and may enter the body through indoor dust, air, soil, water, and the food chain. BDE-47 is highly lipophilic, environmentally persistent, and bioaccumulative and has been detected in multiple environmental media and human biological samples, including serum, adipose tissue, and breast milk (1). Previous studies of PBDEs and BDE-47 suggest that exposure may be associated with endocrine disruption, oxidative stress, mitochondrial dysfunction, and reproductive toxicity (2–4). However, the key susceptible cell populations and molecular mechanisms through which BDE-47 affects the male reproductive system remain incompletely understood.

Male infertility is a major global reproductive health problem influenced by genetic factors, lifestyle, metabolic status, and environmental exposures. As the principal organ responsible for spermatogenesis and androgen synthesis, the testis is particularly sensitive to environmental toxicants and endocrine-disrupting chemicals. Non-obstructive azoospermia (NOA) is one of the most severe forms of male infertility and is primarily caused by intrinsic testicular spermatogenic failure, resulting in markedly impaired or completely absent sperm production. NOA is not restricted to abnormalities in a single cell type but may involve defective germ-cell development, altered Sertoli-cell support, Leydig-cell endocrine dysfunction, and disruption of the local metabolic microenvironment (5). The testis also has a distinctive immune microenvironment, and local immune homeostasis is important for maintaining spermatogenesis and protecting germ cells. Previous evidence suggests that NOA may be accompanied by alterations in immune-cell composition and inflammatory status, which may further disrupt the spermatogenic microenvironment (6, 7). Accordingly, investigating the potential relationship between BDE-47 and NOA from cell-type-specific and immune-microenvironment perspectives may improve our understanding of environmental-pollutant-related male reproductive injury.

Network toxicology integrates bioinformatics, systems biology, and computational toxicology to predict biological pathways and molecular targets potentially perturbed by environmental chemicals through compound-target-disease networks and to provide candidate molecules for subsequent investigation (8). Single-cell RNA sequencing (scRNA-seq), meanwhile, reveals transcriptional heterogeneity among cell types within complex tissues and can localize candidate molecular features associated with disease or toxicant exposure (9). Because the testis is highly heterogeneous and contains multiple germ-cell and somatic-cell populations, integrating network toxicology with single-cell transcriptomics may facilitate the investigation of BDE-47-related reproductive toxicity at both systems and cellular levels (10). In particular, glutathione metabolism, lipid-peroxidation defense, and ferroptosis-related processes may represent important biological components of environmental-toxicant-related testicular injury; therefore, identifying cell-type-specific candidate molecules associated with these processes is of interest (11, 12).

This study aimed to explore candidate relationships among BDE-47 exposure, altered GSTM1 expression in Leydig cells, and ferroptosis-related molecular remodeling using multilayered data. We integrated network toxicology, single-cell data from BDE-47-exposed mouse testes, human idiopathic NOA testicular single-cell data, bulk transcriptomic data, candidate-gene prioritization, molecular simulations, and experiments in TM3 Leydig cells. We constructed and localized a BDE-47-NOA-related candidate gene signature, defined here as a computational overlap among BDE-47-related targets, NOA-associated genes, and BDE-47-exposure-related single-cell differentially expressed genes rather than an established causal relationship between BDE-47 exposure and human NOA. We assessed the consistency of its distribution across mouse and human testicular cell atlases and further explored associations between GSTM1 expression and estimated immune-cell composition in NOA tissue.

## 2 Materials and methods

### 2.1 Acquisition of BDE-47 chemical information

Chemical information for BDE-47 was obtained from PubChem (https://pubchem.ncbi.nlm.nih.gov/), including its two-dimensional and three-dimensional structures, canonical SMILES, molecular formula, molecular weight, and other basic physicochemical properties. These structural data were used for subsequent toxicity-feature prediction, potential-target collection, molecular docking, and compound-related analyses.

### 2.2 Prediction of BDE-47 toxicity features

To preliminarily evaluate the potential toxicity profile of BDE-47, its canonical SMILES was submitted to ProTox 3.0 (https://tox.charite.de/). Predictions included acute toxicity, organ toxicity, toxicological endpoints, toxicity-related molecular targets, and associated biological pathways. The predicted outcomes, probabilities, and confidence scores returned by the platform were organized and visualized to summarize potential ecotoxicity, endocrine-receptor-related activity, mitochondrial dysfunction, and other toxicity endpoints associated with BDE-47.

### 2.3 Prediction of potential BDE-47 targets

BDE-47-related genes were collected from the Comparative Toxicogenomics Database (CTD; accessed June 15, 2026) and SwissTargetPrediction (accessed June 15, 2026). CTD initially returned 4,672 BDE-47-associated genes, of which 850 genes with an Interaction Count > 1 were retained. SwissTargetPrediction yielded 100 predicted targets with the species restricted to Homo sapiens. After gene-symbol standardization and deduplication, the two sources collectively yielded 927 BDE-47-related candidate genes. CTD-derived chemical-gene associations and SwissTargetPrediction-derived computational targets were treated as complementary but distinct evidence sources and were not interpreted as experimentally validated direct targets.

### 2.4 Collection of NOA-related genes

NOA-associated genes were obtained from GeneCards (accessed June 15, 2026) using the search term “non-obstructive azoospermia.” A total of 947 genes were retrieved after gene-symbol standardization and deduplication. No additional relevance-score threshold was applied to retain broad candidate coverage for this hypothesis-generating analysis; accordingly, this gene set was interpreted as an inclusive disease-associated candidate set rather than a high-confidence causal gene set.

### 2.5 Identification of shared candidate genes between BDE-47 and NOA

The 927 BDE-47-related candidate genes were intersected with the 947 NOA-associated genes in R, yielding 113 shared candidate genes. The intersection was visualized using a Venn diagram, and this broad shared candidate set was used for subsequent protein-protein interaction network construction, functional enrichment analysis, and single-cell analyses.

### 2.6 PPI network construction and functional enrichment analysis

The shared candidate genes between BDE-47 and NOA were uploaded to STRING (https://string-db.org/) to construct a protein-protein interaction (PPI) network. The species was set to *Homo sapiens*, the minimum interaction confidence was set to 0.400, and isolated or disconnected nodes were retained. Network data were exported and imported into Cytoscape for visualization of potential interactions among candidate proteins. A compound-target-disease network was also constructed using BDE-47, the shared candidate genes, and NOA.

Gene Ontology (GO) and Kyoto Encyclopedia of Genes and Genomes (KEGG) enrichment analyses were performed using the R packages clusterProfiler and org.Hs.eg.db. GO analysis included biological process, cellular component, and molecular function categories. P values were adjusted for multiple testing using the Benjamini-Hochberg method, and an adjusted P value < 0.05 was considered statistically significant. Significant enrichment results were visualized using ggplot2 and related R packages.

### 2.7 Processing of single-cell data from BDE-47-exposed mouse testes and an external normal reference

Single-cell RNA-sequencing data from BDE-47-exposed mouse testes were obtained from OMIX792. The BDE-47-exposed samples were used to construct the exposed mouse testicular single-cell atlas. According to the original study, C57BL/6J mice received intraperitoneal BDE-47 at 10 mg/kg/day for 3 consecutive weeks beginning on postnatal day 22. The normal mouse testicular single-cell dataset GSE216343 was used solely as an external normal reference atlas to verify major testicular cell types and describe cell-type distribution in normal mouse testes (13, 14).

OMIX792 and GSE216343 were independently subjected to quality control, normalization, dimensionality reduction, clustering, and cell annotation. The two datasets were not jointly batch-corrected and were not used for direct differential-expression analysis or statistical comparison of cell proportions. Expression matrices were separately imported into Seurat objects. Cells with 200-10,000 detected genes, <20% mitochondrial transcripts, and <20% ribosomal transcripts were retained. Following quality control, data were normalized using LogNormalize, followed by highly variable gene selection, scaling, principal component analysis (PCA), nearest-neighbor graph construction, clustering, and uniform manifold approximation and projection (UMAP). Cell types were manually annotated according to cluster marker genes and established testicular cell markers.

BDE-47-related cell-type-specific differentially expressed genes were obtained directly from the Supplementary Materials of the original OMIX792 study, in which the differential-expression results were generated from internal batch-matched comparisons (13). Mouse genes were converted to their human orthologs for subsequent construction of the candidate signature. These genes were not recalculated through a cross-study comparison between OMIX792 and GSE216343.

### 2.8 Processing and annotation of human NOA testicular single-cell data

Human NOA testicular scRNA-seq data were obtained from GSE149512. Five adult control samples and three idiopathic non-obstructive azoospermia (iNOA) samples were included. Raw count matrices were imported into Seurat for quality control. Cells with 200-10,000 detected genes, <20% mitochondrial transcripts, and <20% ribosomal transcripts were retained. The filtered data were normalized using LogNormalize, followed by highly variable gene selection, scaling, and PCA. Harmony was then used to correct sample-level batch effects, and nearest-neighbor graph construction, clustering, and UMAP visualization were performed in the corrected low-dimensional space. Cell clusters were manually annotated using marker genes identified by FindAllMarkers and established testicular cell markers.

### 2.9 Scoring of the BDE-47-NOA-related candidate signature and localization of candidate cell populations

To localize BDE-47-NOA-related candidate molecular features within testicular cell atlases, potential BDE-47-related targets, NOA-related genes, and BDE-47-exposure-related single-cell differentially expressed genes from mice were intersected to construct a candidate gene signature. Here, this signature denotes a computational overlap used for exploratory cellular-context localization rather than evidence of human BDE-47 causality or biological susceptibility. Mouse genes were converted to their corresponding human orthologs before intersection analysis.

UCell was used to calculate cell-level candidate-signature scores in the OMIX792 BDE-47-exposed mouse testicular single-cell data and the human iNOA testicular single-cell data. Score distributions across testicular cell types were compared separately within each dataset. The GSE216343 external normal reference was not used for candidate-signature scoring or between-group score comparisons. This analysis was intended for cell-type localization of candidate molecular features and assessment of cross-species consistency in cell-type distribution; it was not used to establish biological susceptibility.

### 2.10 hdWGCNA of Leydig cells

Leydig cells were extracted from the human testicular single-cell dataset, and high-dimensional weighted gene co-expression network analysis (hdWGCNA) was used to construct a gene co-expression network. Cells with similar transcriptional profiles were first aggregated into metacells to reduce sparsity and technical noise, after which co-expression gene modules were identified in Leydig cells.

To evaluate the cell-type distribution of each module, module eigengene values were projected onto the complete testicular single-cell atlas, and module activity and expression fractions were compared across cell types. The module showing the most prominent activity and expression fraction in Leydig cells was defined as the Leydig cell-enriched module. KEGG enrichment analysis was then performed on genes in this module to characterize the biological processes represented by the Leydig-cell-associated co-expression program.

### 2.11 Metacell-based exploratory machine learning and SHAP candidate-gene prioritization

To further prioritize candidate molecules associated with NOA-related molecular features in Leydig cells, an exploratory single-cell machine-learning analysis was performed using the adult-control or iNOA origin of human Leydig cells as the classification label. Transcriptionally similar cells were aggregated into metacells using the hdWGCNA workflow to reduce sparsity and technical noise. Each metacell contained 35 cells, and 500 metacells were targeted for each group. The normalized metacell expression matrix was then matched to the 113 shared candidate genes identified from the intersection of BDE-47-related candidate genes and NOA-associated genes. Genes with a mean expression <0.01 or an expression variance <0.001 were excluded before subsequent feature-ranking analysis. Metacells derived from the same donor were not considered independent biological replicates; therefore, this analysis was used only for exploratory feature prioritization.

Nine methods were used for feature ranking: random forest, extreme gradient boosting (XGBoost), least absolute shrinkage and selection operator (LASSO) regression, ridge regression, elastic net, radial-kernel support vector machine, linear support vector machine, gradient boosting machine, and naive Bayes. The top 30 genes from each method were retained. These ranking methods were then combined with five classifiers-random forest, radial-kernel support vector machine, XGBoost, elastic net, and naive Bayes-to construct 45 algorithm combinations. Each combination underwent repeated 10-fold cross-validation with 10 repeats for exploratory internal evaluation. Internal model metrics were used only for exploratory comparison among algorithm combinations and were not interpreted as donor-independent predictive performance or clinical diagnostic performance.

Candidate genes were prioritized according to their frequency across algorithms and their SHapley Additive exPlanations (SHAP) contributions. Genes selected in at least 16 of the 45 algorithm combinations were retained as consensus candidates. A random-forest model and SHAP analysis were then used to describe the relative contribution of candidate genes to the adult-control versus iNOA classification output. GSTM1 was selected for downstream exploratory analysis through integrative evidence convergence, including repeated selection across algorithms, relatively prominent SHAP contribution, expression consistency across evidence layers, and redox-detoxification biological plausibility, rather than statistical superiority based on a single ranking metric. Other consensus candidates were not excluded as potentially biologically relevant genes.

### 2.12 GSTM1 expression grouping and ferroptosis-driver gene signature scoring

Leydig cells were extracted from the three iNOA samples and were divided, within the iNOA group only, into GSTM1-high and GSTM1-low groups according to the median GSTM1 expression level. A cell-level ferroptosis-driver gene signature score was then calculated using the ferroptosis-driver gene set collected in FerrDb (https://www.zhounan.org/ferrdb/). Scores were compared between the two Leydig-cell groups to explore the relationship between GSTM1 expression status and ferroptosis-related molecular features in iNOA Leydig cells. This comparison was considered an exploratory cell-level association analysis rather than a donor-level biological inference.

### 2.13 Bulk transcriptomic comparison and exploratory immune deconvolution

The GSE145467 bulk testicular transcriptomic dataset was downloaded from the Gene Expression Omnibus (GEO; https://www.ncbi.nlm.nih.gov/geo/). The dataset included 10 obstructive azoospermia (OA) samples with preserved spermatogenesis and 10 NOA samples with spermatogenic failure. The OA samples were used as normal-spermatogenesis controls. After normalization and probe annotation, GSTM1 expression was compared between normal-spermatogenesis controls and NOA samples and visualized using box plots.

CIBERSORT was used for exploratory deconvolution to estimate the relative composition of immune-cell types in each sample, using the LM22 signature matrix, 100 permutations, and quantile normalization. Samples with a CIBERSORT P value <0.05 were retained for downstream correlation analyses. Associations between GSTM1 expression and estimated immune-cell fractions were evaluated using Spearman correlation, and P values across immune-cell components were adjusted using the Benjamini-Hochberg method. This analysis was intended only to explore potential statistical relationships between GSTM1 expression and estimated immune-cell composition in NOA testicular tissue.

### 2.14 Molecular docking, two-dimensional interaction analysis, and molecular dynamics simulation

To evaluate potential structural compatibility between BDE-47 and GSTM1, the three-dimensional structure of BDE-47 was obtained from PubChem and energy-minimized, whereas the crystal structure of GSTM1 was obtained from the RCSB Protein Data Bank (PDB ID: 7BEU). The complete downloaded 7BEU PDB coordinate file was retained for docking and simulation, and no individual protein chain was extracted. Crystallographic water molecules and nonessential ligands were removed from the protein structure, followed by hydrogen addition and charge assignment. Blind docking was performed using AutoDock Vina with default settings, with the docking grid covering the entire protein structure. The top-ranked pose was selected for subsequent molecular dynamics simulation and was imported into Discovery Studio for two-dimensional interaction analysis.

All-atom molecular dynamics simulations of the BDE-47-GSTM1 complex were performed using GROMACS. The AMBER99SB force field was used for the protein, the General Amber Force Field (GAFF) was used for ligand parameterization, and the TIP3P model was used for water. The complex was placed in a cubic water box, and sodium and chloride ions were added to neutralize the system. Following energy minimization, equilibration was sequentially performed under constant number of particles, volume, and temperature (NVT) conditions and constant number of particles, pressure, and temperature (NPT) conditions. Temperature and pressure were controlled using the V-rescale thermostat and Parrinello–Rahman barostat, respectively, with an integration time step of 2 fs; long-range electrostatic interactions were treated using the particle-mesh Ewald method. A 100-ns production simulation was then conducted at 310 K and 1 bar.

After simulation, the trajectory was analyzed for the root-mean-square deviation (RMSD) of the complex, root-mean-square fluctuation (RMSF) of protein residues, radius of gyration (Rg), solvent-accessible surface area (SASA), number of protein-ligand hydrogen bonds, and free-energy landscape to assess the overall conformational stability and dynamic characteristics of the BDE-47-GSTM1 complex. Trajectory stability was assessed from the temporal behavior of RMSD, Rg, and SASA and was not interpreted as evidence of confirmed binding convergence.

### 2.15 TM3 mouse Leydig-cell culture, experimental treatment, and selection of the working concentration

TM3 mouse Leydig cells were purchased from Cellverse (catalog no. iCell-m058) and cultured in Dulbecco’s modified Eagle medium (DMEM) supplemented with 10% fetal bovine serum and 1% penicillin-streptomycin at 37 °C in a humidified atmosphere containing 5% CO_2_. BDE-47 was purchased from AbMole Bioscience (catalog no. M55229; CAS no. 5436-43-1; purity ≥97%), and ferrostatin-1 was purchased from AbMole Bioscience (catalog no. M2698; CAS no. 347174-05-4). BDE-47 and Fer-1 stock solutions were prepared in dimethyl sulfoxide (DMSO) and diluted to the required working concentrations in complete culture medium. The final DMSO concentration was ≤0.1% (v/v) and was matched across vehicle and treatment groups.

For concentration-gradient experiments, TM3 cells were exposed to 5, 10, or 20 μM BDE-47 for 24 h. GSTM1 protein expression was assessed after treatment with 5 or 10 μM BDE-47 for 24 h.

Ferrostatin-1 (Fer-1) was used as a pharmacological inhibitor in the ferroptosis-related intervention experiment (15). The BDE-47 group received 10 μM BDE-47 for 24 h, and the Fer-1 group received 0.5 μM Fer-1 for the final 12 h of the corresponding treatment window. For the BDE-47 + Fer-1 group, cells were first exposed to 10 μM BDE-47 for 12 h; 0.5 μM Fer-1 was then added without changing the medium or removing BDE-47, and the cells were co-incubated for an additional 12 h. The final DMSO concentration was matched across groups.

The working concentration of BDE-47 was selected according to concentration-gradient pilot experiments. Compared with the DMSO vehicle control, 10 μM BDE-47 for 24 h consistently reduced TM3-cell metabolic activity and GSTM1 protein expression, while producing a smaller decrease in cellular metabolic activity than 20 μM BDE-47. Therefore, 10 μM BDE-47 was selected as the experimental nominal working concentration for subsequent Fer-1 intervention and ferroptosis-related gene-expression experiments. This nominal micromolar concentration was selected to obtain a reproducible molecular response and was not interpreted as a direct surrogate for human or intratesticular exposure.

### 2.16 CCK-8 assay of cellular metabolic activity

TM3 cells were seeded in 96-well plates and, after attachment, were exposed to 5, 10, or 20 μM BDE-47 for 24 h. An untreated control and a vehicle-control group containing the same final concentration of DMSO as the BDE-47-treated groups were included. After treatment, Cell Counting Kit-8 reagent was added according to the manufacturer’s instructions, and cells were incubated at 37 °C in the dark before absorbance was measured at 450 nm. Cellular metabolic activity was normalized to the DMSO vehicle-control group. Experiments were independently repeated at least three times.

### 2.17 Western blotting of GSTM1

After treatment, total protein was extracted from TM3 cells using radioimmunoprecipitation assay (RIPA) lysis buffer containing protease inhibitors, and protein concentrations were determined using a bicinchoninic acid (BCA) assay. Equal amounts of protein were separated by sodium dodecyl sulfate-polyacrylamide gel electrophoresis (SDS-PAGE) and transferred to polyvinylidene fluoride (PVDF) membranes. After blocking with 5% nonfat milk, the membranes were incubated overnight at 4 °C with antibodies against GSTM1 (catalog no. 12412-1-AP; 1:20,000) and β-actin (catalog no. 66009-1-Ig; 1:20,000). Both primary antibodies were purchased from Proteintech.

After washing, the membranes were incubated at room temperature with horseradish-peroxidase-conjugated goat anti-mouse immunoglobulin G (IgG; catalog no. RGAM001; 1:10,000) or goat anti-rabbit IgG (catalog no. RGAR001; 1:10,000), as appropriate. Protein bands were detected using enhanced chemiluminescence, and densitometric analysis was performed in ImageJ. GSTM1 protein expression was normalized to β-actin. Experiments were independently repeated at least three times.

### 2.18 RNA extraction and quantitative real-time PCR

After treatment, total RNA was extracted from TM3 cells and reverse-transcribed into complementary DNA (cDNA). The ferroptosis-related intervention experiment included a vehicle-matched control group (CON), a Fer-1 group, a BDE-47 group, and a BDE-47 + Fer-1 group. Quantitative real-time polymerase chain reaction (qPCR) was used to measure the mRNA expression of *Gpx4*, *Acsl4*, and *Slc7a11*. Amplification was performed using gene-specific primers. Relative expression was calculated using the 2^−ΔΔCt method and normalized to an internal reference gene. Each experiment was independently repeated at least three times. The primer sequences were as follows: *Gpx4*, forward 5′-GCCTTCCCGTGTAACCAGTC-3′ and reverse 5′-GGAGATAGCACGGCAGGTC-3′; *Slc7a11*, forward 5′-ATGCTTCCTGGAGCCATTGG-3′ and reverse 5′-CACAGCACTGCCAAGTCGTA-3′; and *Acsl4*, forward 5′-GGGAGACATCTATGCCACCA-3′ and reverse 5′-TTCTCCACCTGCTTCTCCAC-3′; β-actin, forward GATGAGATTGGCATGGCTTT, reverse CACCTTCACCGGTCCAGTTT.

### 2.19 Statistical analysis

Bioinformatic analyses were mainly performed in R. P values from functional enrichment analyses were adjusted for multiple testing using the Benjamini-Hochberg method, and an adjusted P value < 0.05 was considered statistically significant. Two-group comparisons of cell-level scores were performed using the Wilcoxon rank-sum test. Associations between GSTM1 expression and estimated immune-cell fractions were evaluated using Spearman correlation, and P values from correlations across multiple immune-cell components were adjusted using the Benjamini-Hochberg method. Cell-level P values were interpreted as exploratory distributional or association statistics and were not considered donor-level biological inference.

*In vitro* data were analyzed using GraphPad Prism and are presented as the mean ± standard deviation. CCK-8 data were analyzed by one-way analysis of variance (ANOVA) followed by Dunnett’s multiple-comparisons test using the DMSO vehicle-control group as the reference. Multigroup GSTM1 Western blot and qPCR data were analyzed by one-way ANOVA followed by Tukey’s multiple-comparisons test. All *in vitro* experiments were independently repeated at least three times. All tests were two-sided, and P < 0.05 was considered statistically significant.

## 3 Results

### 3.1 Shared candidate genes between BDE-47 and NOA are enriched in oxidative-stress-, ferroptosis-, and reproductive-endocrine-related processes

We first predicted the potential toxicity features of BDE-47. ProTox 3.0 suggested that BDE-47 may be associated with ecotoxicity, endocrine-receptor-related activity, altered mitochondrial membrane potential, cytochrome P450-related metabolism, and other toxicity endpoints (**Figure 1A**). The BDE-47-related candidate set comprised 850 CTD genes with an Interaction Count > 1 and 100 SwissTargetPrediction targets; after gene-symbol standardization and deduplication, 927 BDE-47-related candidate genes were retained. Intersection with 947 NOA-associated genes yielded 113 shared candidate genes (**Figure 1B**).

**Figure 1:**
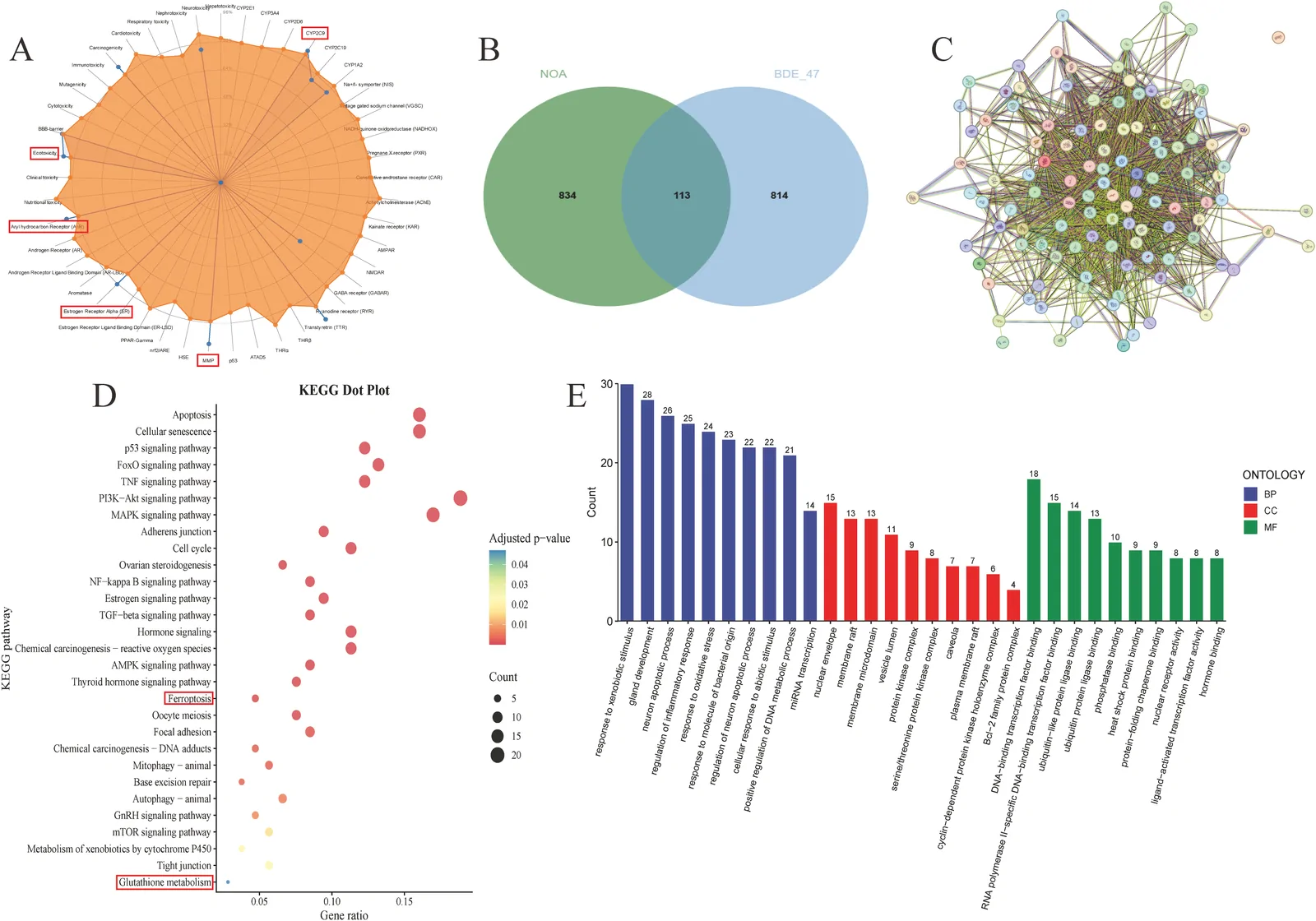
Network-toxicology analysis of shared candidate genes between BDE-47 and NOA. **(A)** BDE-47 toxicity features predicted by ProTox 3.0. **(B)** Intersection of potential BDE-47-related targets and NOA-related genes, yielding 113 shared candidate genes. **(C)** Protein-protein interaction (PPI) network of the shared candidate genes. **(D)** Kyoto Encyclopedia of Genes and Genomes (KEGG) enrichment analysis; dot size represents the number of enriched genes, and color represents the adjusted P value. **(E)** Gene Ontology (GO) enrichment results for biological process (BP), cellular component (CC), and molecular function (MF).

The PPI network constructed from the shared candidate genes showed extensive potential interactions among candidate proteins (**Figure 1C**). KEGG enrichment analysis indicated that these genes were mainly involved in apoptosis, cellular senescence, p53, phosphoinositide 3-kinase-Akt, and mitogen-activated protein kinase signaling, as well as reactive-oxygen-species-related chemical carcinogenesis, ferroptosis, cytochrome-P450-mediated xenobiotic metabolism, and glutathione metabolism (**Figure 1D**).

GO enrichment analysis further indicated that the genes were involved in xenobiotic metabolism, responses to oxidative stress, reproductive and gland development, mitochondrial processes, and hormone binding (**Figure 1E**).

Together, enrichment of the shared candidate set highlighted xenobiotic metabolism, oxidative stress, glutathione metabolism, ferroptosis-related processes, and reproductive endocrine regulation as biological themes shared across BDE-47-related and NOA-associated gene sets.

### 3.2 Leydig cells are one of the somatic cell populations enriched for the candidate signature in BDE-47-exposed mouse testicular data

To describe the cell-type landscape of BDE-47-exposed mouse testes, we analyzed the OMIX792 single-cell dataset. After independent quality control, clustering, and annotation, 19 cell clusters were obtained and annotated as spermatogonia (SPG), pachytene spermatocytes, meiotic-division spermatocytes, round spermatids, acrosomal spermatids, elongating spermatids, Sertoli cells, Leydig cells, macrophages, and telocytes (**Figures 2A, B**). Independent processing of the GSE216343 external normal mouse testicular dataset identified corresponding major testicular cell types. No direct differential-expression or cell-proportion statistical inference was performed between the two datasets.

**Figure 2:**
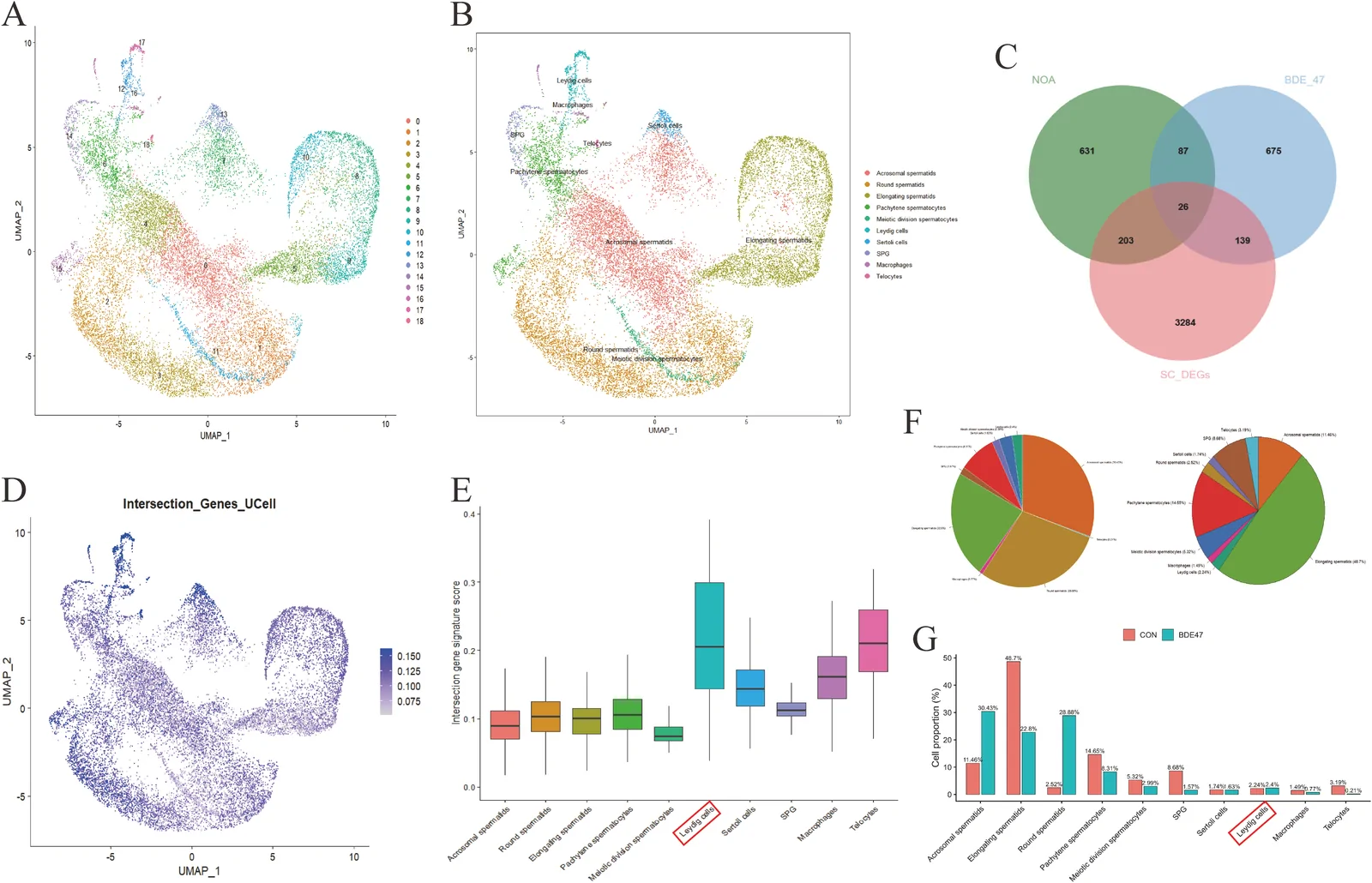
Single-cell atlas of BDE-47-exposed mouse testes and cell-type localization of the candidate signature. **(A)** Unsupervised clustering UMAP of the OMIX792 BDE-47-exposed dataset. **(B)** Annotation of major testicular cell types. **(C)** Three-set intersection of BDE-47-related targets, NOA-related genes, and BDE-47-exposure-related single-cell differentially expressed genes reported in the original study, yielding 26 candidate genes. **(D)** Distribution of the candidate-signature UCell score in the OMIX792 cell atlas. **(E)** Distribution of candidate-signature scores across cell types. **(F, G)** Descriptive cell composition in the independently processed GSE216343 external normal reference and OMIX792 BDE-47-exposed datasets; no direct statistical comparison of cell proportions or causal inference was performed between the datasets. UMAP, uniform manifold approximation and projection.

Potential BDE-47-related targets, NOA-related genes, and BDE-47-exposure-related single-cell differentially expressed genes reported in the Supplementary Materials of the original OMIX792 study were intersected, yielding 26 candidate genes (**Figure 2C**). UCell scoring of this candidate signature in the OMIX792 BDE-47-exposed single-cell atlas showed that the signature was distributed across multiple testicular cell populations (**Figure 2D**), with Leydig cells showing relatively high scores and representing one of the somatic cell populations enriched for the signature (**Figure 2E**). Leydig cells were therefore selected as the focus of subsequent candidate-molecule analyses and *in vitro* validation. **Figures 2F, G** are therefore presented only as descriptive cell-composition summaries and were not used to infer BDE-47-related changes in Leydig-cell abundance, preferential susceptibility, or functional impairment.

### 3.3 Human single-cell analysis and exploratory candidate-gene ranking highlight GSTM1

To evaluate cross-species consistency in the cell-type distribution of the candidate signature, we analyzed the GSE149512 human testicular single-cell dataset. Following dimensionality reduction, clustering, and annotation, major cell populations were identified, including peritubular myoid (PTM) cells, Leydig cells, spermatids (SPTs), spermatocytes (SPCs), spermatogonia (SPGs), Sertoli cells, macrophages, mast cells, endothelial cells, and vascular smooth muscle (VSM) cells (**Figures 3A, B**).

**Figure 3:**
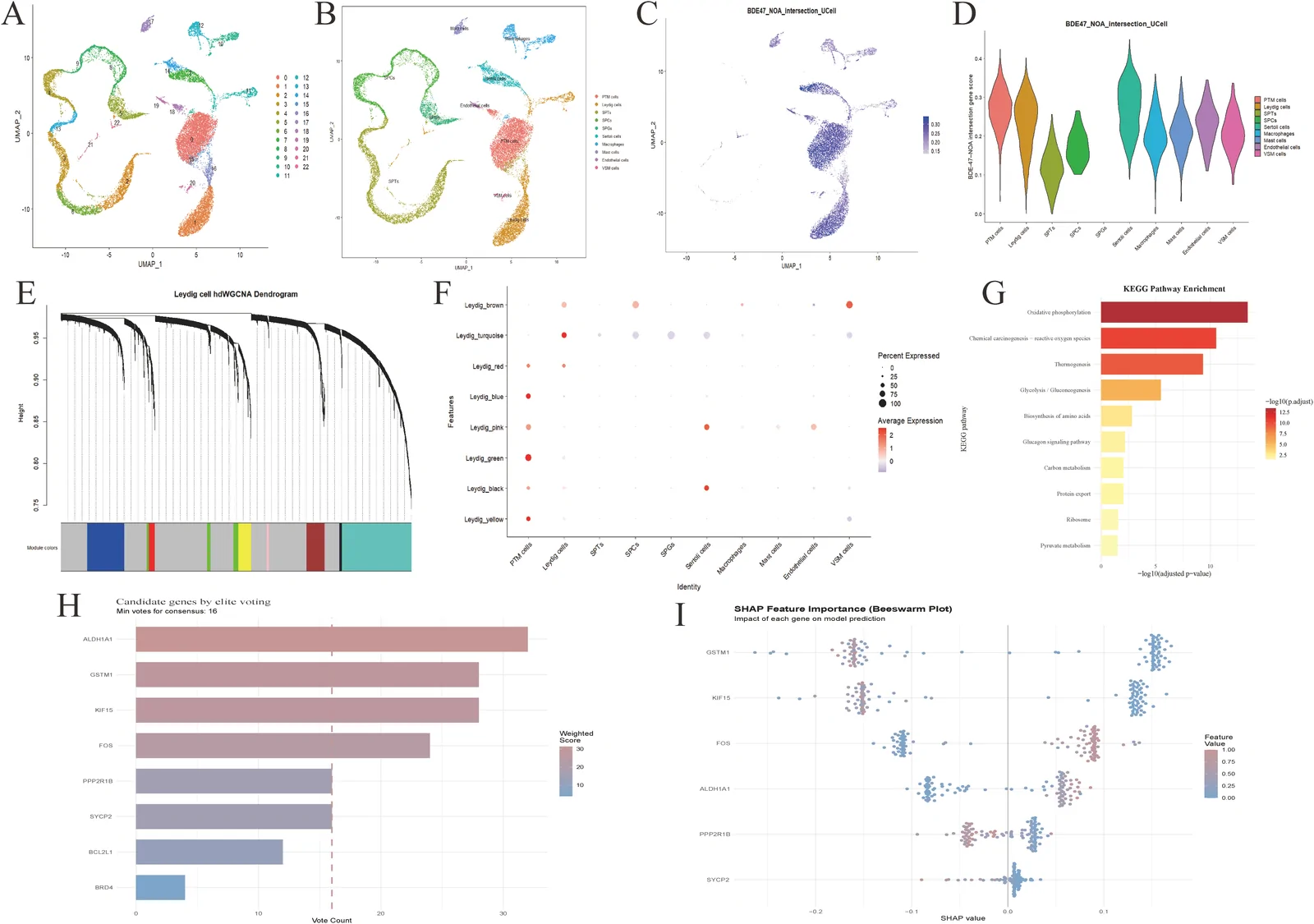
Human testicular single-cell analysis, Leydig-cell hdWGCNA, and exploratory candidate-gene ranking. **(A)** Unsupervised clustering UMAP of human testicular single-cell data. **(B)** Annotation of major cell types. **(C)** Distribution of the BDE-47-NOA candidate-signature UCell score. **(D)** Candidate-signature scores across cell types. **(E)** Leydig-cell hdWGCNA dendrogram and module assignment. **(F)** Mean module expression and expressing-cell fraction across testicular cell types. **(G)** KEGG enrichment analysis of the Leydig cell-enriched turquoise module. **(H)** Candidate genes retained by elite voting; the dashed line indicates the prespecified voting threshold. **(I)** SHAP beeswarm plot from the random-forest model, showing the relative contribution of candidate genes to model output. hdWGCNA, high-dimensional weighted gene co-expression network analysis; SHAP, SHapley Additive exPlanations.

After projection of the BDE-47-NOA-related candidate signature onto the human testicular single-cell atlas, the signature showed varying degrees of enrichment across multiple cell populations (**Figures 3C, D**), and Leydig cells remained one of the higher-scoring somatic cell types. This finding supported a degree of consistency in cell-type distribution between the mouse and human datasets but did not constitute independent mechanistic validation.

Leydig cells were then extracted from the human single-cell dataset for hdWGCNA, which identified multiple co-expression modules (**Figure 3E**). After projection of module features onto the complete testicular atlas, the turquoise module showed relatively prominent module activity and expression fraction in Leydig cells and was therefore defined as the Leydig cell-enriched module (**Figure 3F**).

KEGG enrichment analysis showed that genes in the turquoise module were mainly involved in oxidative phosphorylation, chemical carcinogenesis-reactive oxygen species, thermogenesis, glycolysis/gluconeogenesis, carbon metabolism, protein export, ribosome, and pyruvate metabolism, suggesting that this module mainly represented co-expression programs related to energy metabolism, oxidative stress, and protein synthesis in Leydig cells (**Figure 3G**).

Starting from the 113 shared candidate genes, a metacell-based exploratory feature-prioritization analysis was subsequently performed using adult-control versus iNOA status as the classification label. Using the prespecified voting threshold, ALDH1A1, GSTM1, KIF15, FOS, PPP2R1B, and SYCP2 were retained as consensus candidate genes (**Figure 3H**). Relative SHAP contributions from the random-forest model are shown in **Figure 3I**. GSTM1 was selected for downstream exploratory analysis based on repeated selection across algorithms, relatively prominent SHAP contribution, concordant expression across BDE-47-related and human NOA-associated evidence layers, and glutathione/redox biological plausibility. This integrative prioritization did not indicate statistical superiority over the other consensus candidates or donor-independent predictive performance.

### 3.4 Low GSTM1 expression is associated with the Leydig-cell ferroptosis-driver gene signature and estimated immune-cell composition in NOA tissue

To further characterize heterogeneity in GSTM1 expression within iNOA Leydig cells, Leydig cells were extracted from the human testicular atlas and reclustered (**Figures 4A, B**). GSTM1 showed heterogeneous expression across Leydig-cell subclusters (**Figure 4C**). Cells from the three iNOA samples were then divided, within iNOA only, into GSTM1-high and GSTM1-low groups according to the median GSTM1 expression level (**Figure 4D**). At the cell level, GSTM1-low Leydig cells had a higher ferroptosis-driver gene signature score than GSTM1-high cells (**Figure 4E**), indicating an exploratory association between lower GSTM1 expression and a stronger ferroptosis-related transcriptional signature in iNOA Leydig cells.

**Figure 4:**
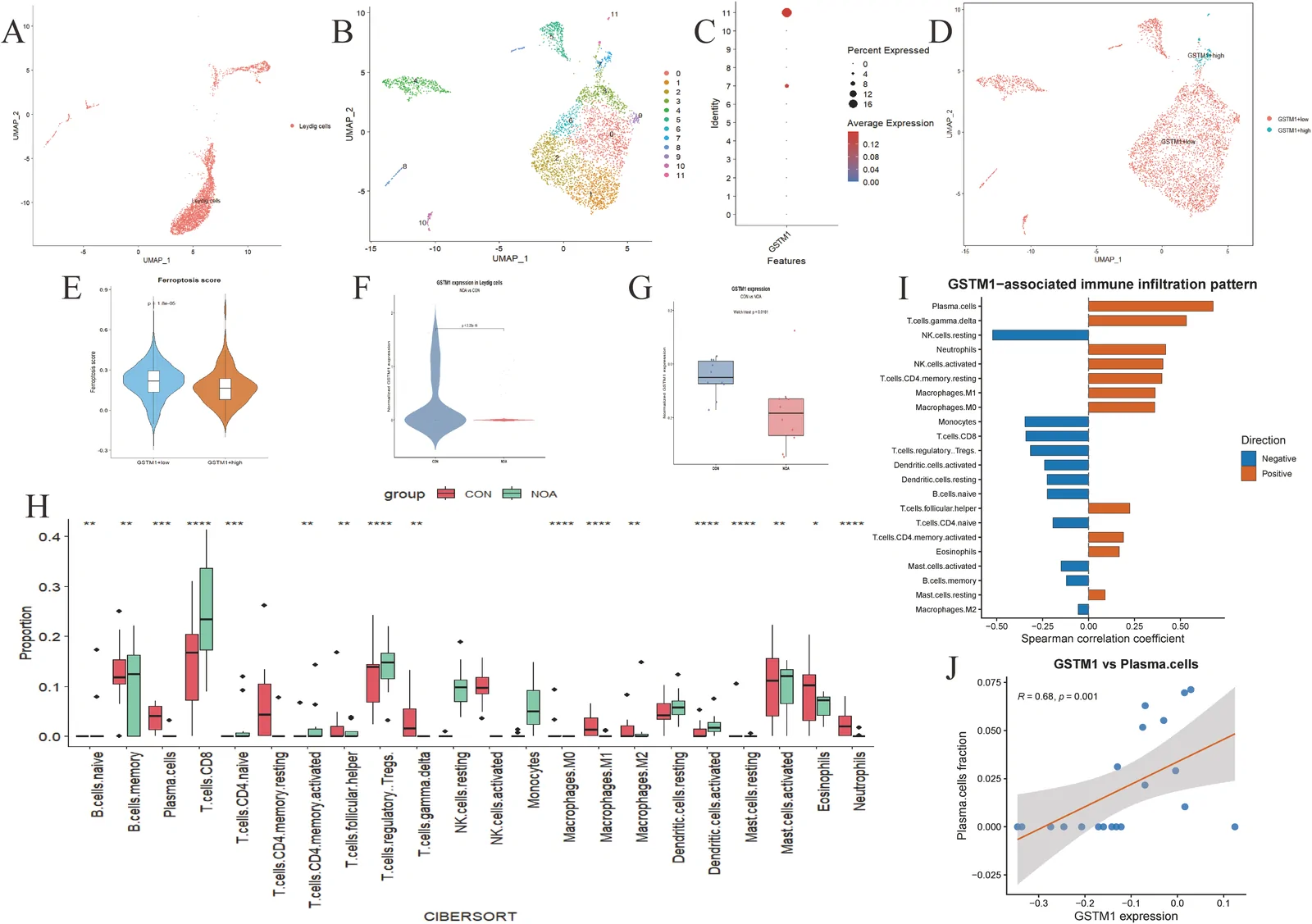
Associations among GSTM1 expression, the ferroptosis-driver gene signature, and estimated immune-cell composition. **(A)** Leydig cells extracted from the human testicular atlas. **(B)** Reclustering of Leydig cells. **(C)** GSTM1 expression across Leydig-cell subclusters. **(D)** GSTM1-high and GSTM1-low grouping of iNOA Leydig cells. **(E)** Ferroptosis-driver gene signature scores in the two groups. **(F)** GSTM1 expression in adult-control and iNOA Leydig cells. **(G)** GSTM1 expression in normal-spermatogenesis controls and NOA tissue in GSE145467. **(H)** Relative immune-cell composition estimated by CIBERSORT. **(I)** Spearman correlation coefficients between GSTM1 expression and estimated immune-cell fractions. **(J)** Correlation between GSTM1 expression and the estimated plasma-cell fraction. Immune-deconvolution and correlation results are exploratory. iNOA, idiopathic non-obstructive azoospermia.

We next described GSTM1 expression across mouse BDE-47 exposure data, human single-cell data, and bulk transcriptomic data. In the internal comparison reported in the Supplementary Materials of the original OMIX792 study, *Gstm1* was identified as a downregulated gene in BDE-47-exposed Leydig cells. In GSE149512, GSTM1 expression was lower in iNOA Leydig cells than in adult-control Leydig cells (**Figure 4F**). The GSE145467 bulk transcriptomic dataset likewise showed lower GSTM1 expression in NOA samples than in OA controls with preserved spermatogenesis (**Figure 4G**). These findings indicated descriptive consistency in the direction of GSTM1 expression changes between the mouse-exposure and human-NOA datasets.

CIBERSORT analysis indicated multiple differences in estimated immune-cell composition between normal-spermatogenesis controls and NOA tissue (**Figure 4H**). Correlation analysis further showed positive or negative exploratory associations between GSTM1 expression and several estimated immune-cell fractions (**Figure 4I**). Among these components, plasma cells showed the strongest positive association with GSTM1. The estimated plasma-cell fraction was also lower in NOA than in control tissue, paralleling the lower GSTM1 expression observed in NOA (**Figure 4J**). Because OA and NOA testes differ markedly in germ-cell composition, these relative immune-cell estimates may be influenced by broader tissue-compositional differences and do not imply a direct biological or mechanistic relationship.

Collectively, at the cell level, low GSTM1 expression was associated with a higher ferroptosis-driver gene signature score in Leydig cells and with variation in estimated immune-cell composition in NOA testicular tissue. These exploratory observations do not establish direct regulation of ferroptosis-related processes or immune-cell composition by GSTM1.

### 3.5 Molecular simulations suggest potential structural compatibility between BDE-47 and GSTM1, while *in vitro* experiments show GSTM1 reduction and transcriptional changes consistent with a ferroptosis-related pattern

Molecular docking generated a predicted BDE-47-GSTM1 pose with a binding energy of -6.6 kcal/mol. Two-dimensional interaction analysis suggested that the pocket was formed by or adjacent to TYR6, TRP7, ILE9, LEU12, ARG42, ASN58, LEU59, HIS107, MET108, GLY111, MET112, TYR115, PHE208, and SER209, with several residues potentially contributing hydrophobic and other noncovalent interactions (**Figure 5A**).

**Figure 5:**
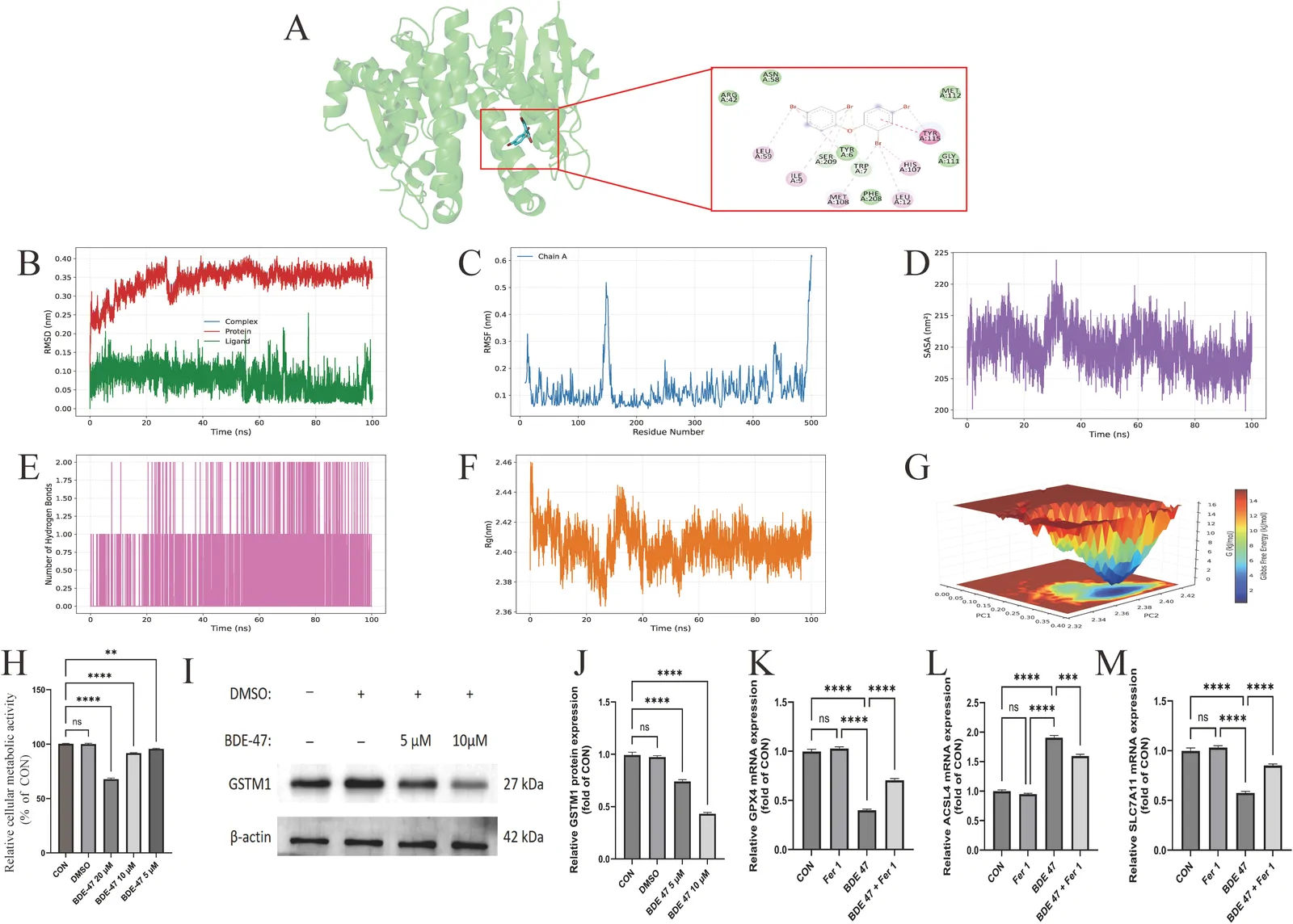
Molecular simulation of BDE-47 and GSTM1 and *in vitro* evaluation in TM3 cells. **(A)** Computationally predicted structural pose of BDE-47 with GSTM1 and two-dimensional interaction pocket. **(B)** Root-mean-square deviation (RMSD) of the complex, protein, and ligand. **(C)** Root-mean-square fluctuation (RMSF) of protein residues. **(D)** Solvent-accessible surface area (SASA). **(E)** Number of protein-ligand hydrogen bonds. **(F)** Radius of gyration (Rg). **(G)** Free-energy landscape. **(H)** Relative cellular metabolic activity after treatment with 5, 10, or 20 μM BDE-47 for 24 h. **(I)** Representative Western blot bands for GSTM1 and β-actin. **(J)** Densitometric quantification of GSTM1 protein expression. **(K–M)** Relative mRNA expression of *Gpx4*, *Acsl4*, and *Slc7a11* following Fer-1 intervention. BDE-47 decreased *Gpx4* and *Slc7a11* and increased *Acsl4*, whereas Fer-1 partially attenuated these changes. Data are presented as the mean ± standard deviation; statistical methods are described in the Materials and methods. ns, not significant; *P < 0.05; **P < 0.01; ***P < 0.001; ****P < 0.0001.

During the 100-ns molecular dynamics simulation, the complex underwent an initial conformational adjustment and subsequently showed relatively stable overall fluctuations (**Figure 5B**). RMSF analysis identified regions of relatively high flexibility (**Figure 5C**), whereas SASA and Rg did not show a sustained increasing trend (**Figures 5D, F**). The number of protein-ligand hydrogen bonds remained low and hydrogen bonds appeared intermittently during portions of the simulation (**Figure 5E**). The free-energy landscape showed a concentrated conformational distribution within a relatively low-energy region (**Figure 5G**). These results provided computational support for potential structural compatibility between BDE-47 and GSTM1 but did not establish direct binding.

The CCK-8 assay showed no significant difference between the untreated control and DMSO vehicle-control groups. Compared with the DMSO vehicle control, exposure to 5, 10, or 20 μM BDE-47 for 24 h progressively reduced TM3-cell metabolic activity, showing an overall concentration-dependent trend. The 10 μM group showed an approximately 10% reduction, whereas the 20 μM group showed a more pronounced decrease (**Figure 5H**). Therefore, 10 μM BDE-47 was selected as the working concentration for subsequent Fer-1 intervention experiments.

Western blotting showed that DMSO alone did not markedly affect GSTM1 protein expression. Compared with the DMSO vehicle control, both 5 and 10 μM BDE-47 reduced GSTM1 protein expression, with a greater reduction at 10 μM, indicating a concentration-related trend (**Figures 5I, J**). This direction was consistent with the downregulation of *Gstm1* in BDE-47-exposed Leydig cells reported in the Supplementary Materials of the original OMIX792 study.

qPCR showed that, compared with the vehicle-matched control, 10 μM BDE-47 significantly decreased *Gpx4* and *Slc7a11* mRNA expression and increased *Acsl4* mRNA expression. Fer-1 alone did not markedly alter the expression of these genes. In cells exposed to BDE-47, Fer-1 partially restored the reduced expression of *Gpx4* and *Slc7a11* and partially attenuated the increase in *Acsl4* expression (**Figures 5K–M**). The direction of these changes was consistent with a commonly described ferroptosis-related molecular pattern.

In summary, BDE-47 reduced TM3-cell metabolic activity and GSTM1 protein expression and was accompanied by ferroptosis-related transcriptional changes characterized by decreased Gpx4 and Slc7a11 and increased Acsl4. Fer-1 partially attenuated these transcriptional changes, providing limited pharmacological support for the sensitivity of this transcriptional pattern to a ferroptosis-directed intervention. Because only mRNA levels of the ferroptosis-related genes were measured, these findings do not establish ferroptotic cell death.

## 4 Discussion

BDE-47 is a representative congener of PBDE flame retardants and was widely used in electronic equipment, construction materials, furniture, textiles, and other consumer products to reduce material flammability. Because PBDEs are not chemically bound to product matrices, BDE-47 can be gradually released during product manufacturing, use, aging, and disposal and can enter the human body through air, indoor dust, soil, water, and the food chain. Its high lipophilicity, environmental persistence, and bioaccumulation make it difficult to remove completely through conventional environmental treatment, and it continues to be detected in various environmental media and human serum, adipose tissue, and breast milk (1). Consequently, the long-term environmental persistence and human exposure associated with BDE-47 have raised substantial environmental-health concerns. In recent years, its reproductive toxicity has attracted increasing attention, and previous studies suggest that BDE-47 may affect testicular function through endocrine disruption, oxidative stress, mitochondrial dysfunction, and altered steroidogenesis (2–4). Micromolar BDE-47 concentrations have previously been used in Leydig-cell and testis-related experimental systems. For example, adult rat Leydig cells have been exposed to 10_-8_–10_-4_ M BDE-47 *in vitro*, and fetal rat testis organ cultures have been exposed to 10 μM BDE-47 (16, 17). These studies provide methodological precedent for the present working concentration but do not establish equivalence to environmentally encountered or intratesticular exposure levels. Nevertheless, the cellular contexts associated with BDE-47-related testicular responses and the candidate molecular features shared with spermatogenic dysfunction remain insufficiently defined. Investigating BDE-47-related reproductive toxicity at cell-type-specific and molecular-network levels is therefore important for understanding its potential risks to male reproductive health (18).

This study integrated network toxicology, single-cell data from BDE-47-exposed mouse testes, human NOA single-cell data, bulk transcriptomic data, hdWGCNA, exploratory machine learning, molecular simulations, and *in vitro* experiments to investigate candidate relationships between BDE-47-related molecular features and male-infertility-associated molecular abnormalities across multiple layers of evidence. Network-toxicology analysis identified 113 shared candidate genes, and mouse and human single-cell analyses localized the candidate signature to Leydig cells as one of the enriched testicular somatic cell populations. Integration of hdWGCNA, exploratory feature prioritization, and SHAP analysis selected GSTM1 as a redox/detoxification-associated candidate molecule. Molecular simulations suggested potential structural compatibility between BDE-47 and GSTM1. In TM3 cells, 10 μM BDE-47 reduced metabolic activity and GSTM1 protein expression, decreased Gpx4 and Slc7a11 mRNA expression, and increased Acsl4 mRNA expression; Fer-1 partially attenuated these transcriptional changes. Overall, these evidence layers indicate molecular convergence around GSTM1 and ferroptosis-related transcriptional remodeling, rather than a demonstrated causal mechanism. The overall integrative framework and principal findings of the present study are summarized in **Figure 6**.

**Figure 6:**
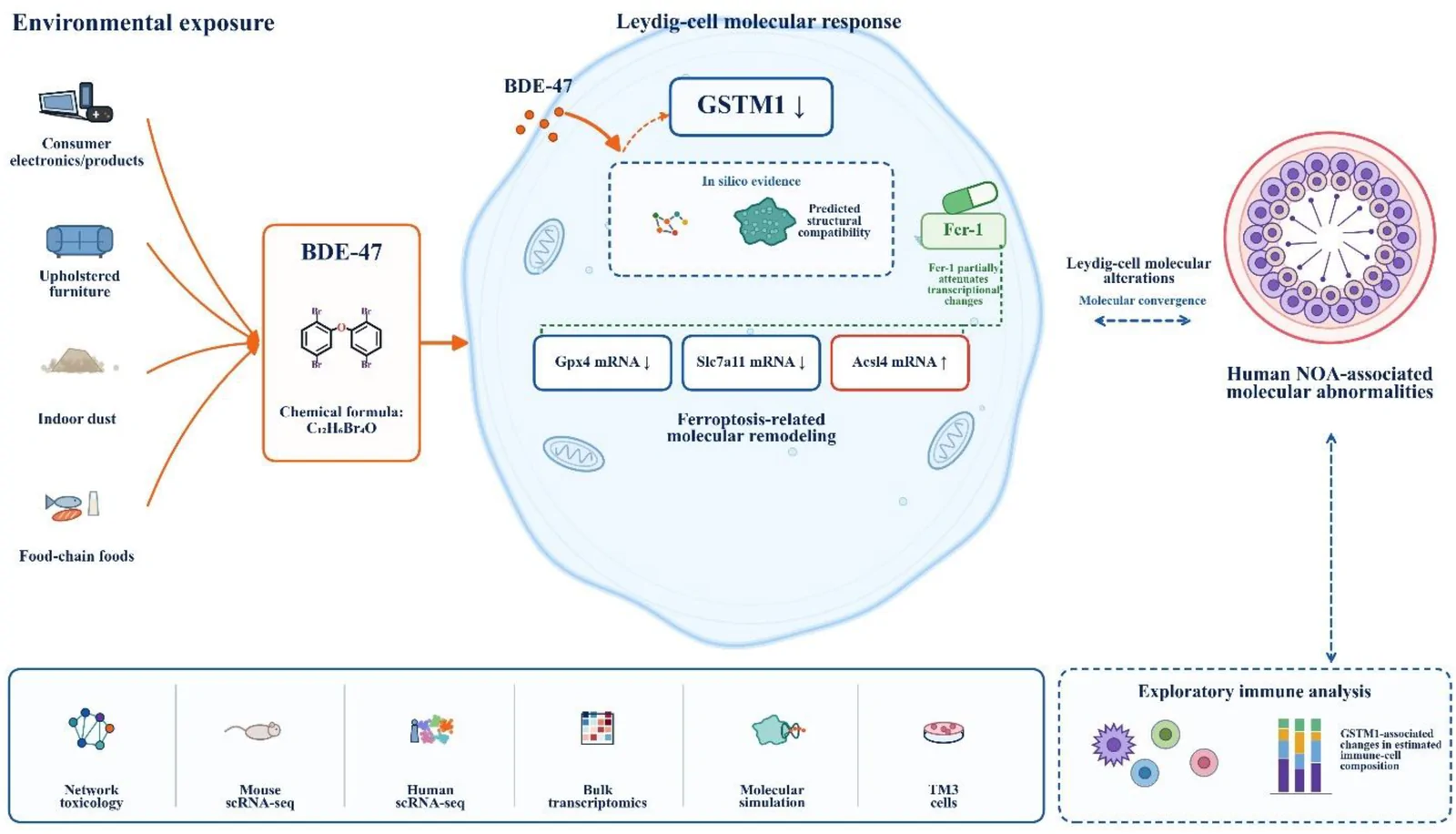
Schematic overview of the study framework and principal findings. The figure summarizes the integrative analysis of BDE-47 exposure, GSTM1-associated Leydig-cell alterations, ferroptosis-related molecular remodeling, and their relevance to human non-obstructive azoospermia.

GSTM1 is an important member of the glutathione S-transferase family and belongs to the mu class of glutathione transferases. It primarily participates in glutathione-dependent xenobiotic metabolism and cellular detoxification. GSTM1 catalyzes the conjugation of reduced glutathione to electrophilic substrates, thereby facilitating the clearance of toxic metabolites, environmental chemicals, and products of oxidative damage. As part of the cellular antioxidant-defense system, GSTM1-related reactions are closely associated with glutathione metabolism, reactive-oxygen-species clearance, detoxification of lipid-peroxidation products, and maintenance of redox homeostasis (19). Previous studies have associated GSTM1 deletion or altered expression with impaired semen quality, increased sperm DNA damage, and greater susceptibility to male infertility, suggesting a potential role in maintaining normal spermatogenesis and testicular function (20–22). Moreover, the glutathione metabolism and lipid-peroxidation detoxification processes involving GSTM1 are closely linked to key ferroptosis-defense mechanisms (23–25), whereas ferroptosis-related alterations have been implicated in spermatogenic dysfunction, Leydig-cell abnormalities, and male infertility (26). Recent evidence further showed that MEHP, the major bioactive metabolite of DEHP, can promote ferroptosis-related injury in Leydig cells through the NOS2/peroxynitrite axis, supporting the broader relevance of ferroptosis-related processes in environmental toxicant-associated Leydig-cell injury (27). However, the molecular context identified in the present study differs in that GSTM1 emerged as a candidate redox and detoxification-associated molecule linking BDE-47-related and NOA-associated molecular features. These established functions provide biological plausibility for selecting GSTM1, but do not establish that GSTM1 mediates the downstream molecular alterations observed after BDE-47 exposure. The exploratory immune-deconvolution results additionally suggested an association between GSTM1 expression and estimated immune-cell composition in NOA testicular tissue, but they do not indicate that GSTM1 directly regulates the immune microenvironment.

Ferroptosis is a regulated form of cell death distinct from apoptosis, necrosis, and pyroptosis and is characterized by the sustained accumulation of iron-dependent lipid peroxides (11, 12). SLC7A11 promotes cystine uptake to maintain glutathione synthesis, GPX4 uses glutathione to reduce lipid hydroperoxides, and ACSL4 promotes the activation of polyunsaturated fatty acids, thereby increasing membrane susceptibility to lipid peroxidation (23–25). In this study, decreased Gpx4 and Slc7a11 mRNA expression and increased Acsl4 mRNA expression were directionally consistent with weakened anti-lipid-peroxidation defense and increased susceptibility to lipid peroxidation. Nevertheless, mRNA expression does not equate to corresponding protein abundance, GPX4 enzymatic activity, or actual lipid-peroxidation status, and the partial attenuation of transcriptional changes by Fer-1 alone cannot establish ferroptotic cell death. These findings are therefore more appropriately interpreted as transcriptional remodeling consistent with a ferroptosis-related pattern rather than evidence that TM3 cells underwent ferroptosis.

The principal strength of this study lies in the convergence of multiple evidence layers and the gradual localization of candidate molecular clues. Rather than relying solely on network-toxicology predictions, we incorporated single-cell data from BDE-47-exposed mouse testes and human iNOA testes to assess cross-species consistency in the distribution of candidate molecular features and to focus the investigation on Leydig cells. hdWGCNA, exploratory feature prioritization, and SHAP analysis provided multidimensional candidate-gene screening and identified GSTM1 as a candidate warranting further validation. Bulk transcriptomic data supported altered GSTM1 expression in human tissue with spermatogenic failure, while molecular docking, molecular dynamics simulation, and TM3-cell experiments provided complementary evidence at the levels of tissue expression, predicted structural compatibility, and cellular molecular response. The Fer-1 intervention showed that BDE-47-associated transcriptional changes were partially attenuated by a ferroptosis-directed compound. This framework provides a systematic, hypothesis-generating basis for further investigation of BDE-47-related male reproductive injury.

Despite these strengths, several limitations should be acknowledged. First, network-toxicology and transcriptomic intersection analyses provide candidate associations and cannot substitute for direct target-binding experiments. The inclusive target-selection strategy may increase candidate coverage while reducing specificity, and no confidence-weighted or stringent-threshold sensitivity analysis was performed. Second, the human NOA datasets did not include individual-level BDE-47 exposure measurements; therefore, they describe NOA-associated molecular alterations rather than BDE-47-induced human alterations. Third, the GSE216343 external normal dataset was used only to independently verify major cell types and assist annotation and was not used for direct differential-expression or cell-proportion comparisons with the OMIX792 BDE-47-exposed data. The mouse-exposure and human-NOA datasets were derived from different species and study contexts, and their cross-species consistency reflects only similarities in the cell-type distribution of candidate molecular features. Metacell-based hdWGCNA may improve network stability but may also reduce representation of cellular heterogeneity. Exploratory machine learning used metacells rather than independent donors as the analytical units, and residual donor-level dependence may have influenced candidate ranking. The GSTM1-high versus GSTM1-low comparison was likewise an exploratory cell-level association analysis and may underestimate inter-donor variation. Molecular docking and a single 100-ns molecular dynamics trajectory support only predicted structural compatibility and do not demonstrate direct target engagement. Immune deconvolution describes associations between GSTM1 expression and estimated immune-cell composition but may be influenced by broader tissue-compositional differences between OA and NOA samples. The CCK-8 assay mainly reflects cellular metabolic activity and cannot distinguish among inhibited proliferation, metabolic suppression, or different forms of cell death. The current experiments measured Gpx4, Acsl4, and Slc7a11 only at the mRNA level and therefore do not reflect corresponding protein abundance or enzymatic activity. Lipid peroxidation, lipid reactive oxygen species, labile iron, and ferroptosis-associated morphology were not directly assessed, and GSTM1 overexpression or knockdown was not performed. The 10 μM nominal medium concentration used in TM3 cells cannot be directly extrapolated to human or intratesticular exposure, particularly given the lipophilicity and protein-binding characteristics of BDE-47. Finally, TM3-cell experiments provide Leydig-cell-level support but cannot substitute for *in vivo* causal validation.

## 5 Conclusion

By integrating network toxicology, single cell transcriptomic data from mouse and human testes, bulk transcriptomic data, hdWGCNA, exploratory feature prioritization, molecular simulations, and *in vitro* experiments, this study identified molecular features shared between BDE-47 exposure and NOA in the context of Leydig cells and prioritized GSTM1 as a candidate molecule related to redox regulation and detoxification. In TM3 cells, BDE-47 exposure was accompanied by reduced GSTM1 expression and transcriptional changes involving ferroptosis related genes, while Fer-1 partially attenuated these changes. Together, these findings provide a basis for further mechanistic investigation of GSTM1 and ferroptosis related molecular alterations in BDE-47 associated male reproductive toxicity, while direct molecular interaction, GSTM1 dependent regulation, ferroptotic cell death, and a causal relationship between BDE-47 exposure and human NOA remain to be established.

## Data Availability

Publicly available datasets were analyzed in this study. The publicly available datasets analyzed in this study were obtained from the OMIX database (https://ngdc.cncb.ac.cn/omix/) under accession number OMIX792 and from the National Center for Biotechnology Information Gene Expression Omnibus database (https://www.ncbi.nlm.nih.gov/geo/) under accession numbers GSE216343, GSE149512, and GSE145467.
